# Modification of Acorn Starch Structure and Properties by High Hydrostatic Pressure

**DOI:** 10.3390/gels9090757

**Published:** 2023-09-17

**Authors:** Luís M. G. Castro, Ana I. Caço, Carla F. Pereira, Sérgio C. Sousa, María E. Brassesco, Manuela Machado, Óscar L. Ramos, Elisabete M. C. Alexandre, Jorge A. Saraiva, Manuela Pintado

**Affiliations:** 1 CBQF-Centro de Biotecnologia e Química Fina—Laboratório Associado, Escola Superior de Biotecnologia, Universidade Católica Portuguesa, Rua Diogo Botelho 1327, 4169-005 Porto, Portugal; lgcastro@ucp.pt (L.M.G.C.); cpfpereira@ucp.pt (C.F.P.); sdsousa@ucp.pt (S.C.S.); mbrassesco@ucp.pt (M.E.B.); mmachado@ucp.pt (M.M.); oramos@ucp.pt (Ó.L.R.); 2 LAQV-REQUIMTE—Laboratório Associado, Department of Chemistry, University of Aveiro, 3810-193 Aveiro, Portugal; elisabete.alexandre.pt@gmail.com (E.M.C.A.); jorgesaraiva@ua.pt (J.A.S.); 3 Laboratório de Análises Térmicas, Department of Chemistry, University of Aveiro, 3810-193 Aveiro, Portugal; icaco@ua.pt

**Keywords:** acorn, starch, extraction, modification, properties, high hydrostatic pressure

## Abstract

Despite being rich in starch, over half of acorn production is undervalued. High hydrostatic pressure was used to modify the properties of *Q. pyrenaica* (0.1 and 460 MPa for 20 min) and *Q. robur* (0.1 and 333 MPa for 17.4 min) acorn starches to obtain high-valued ingredients. Pressure significantly altered the span distribution and heterogeneity of the acorn starch granules depending on the species, but their morphology was unaffected. Pressurization increased the amylose/amylopectin ratio and damaged starch contents, but the effect was more prominent in *Q. pyrenaica* than in *Q. robur*. However, the polymorphism, relative crystallinity, gelatinization temperatures, and enthalpies were preserved. The pressure effect on the starch properties depended on the property and species. The solubility, swelling power, and acorn gels’ resistance towards deformation for both species decreased after pressurization. For *Q. pyrenaica* starch, the in vitro digestibility increased, but the pseudoplastic behavior decreased after pressurization. No differences were seen for *Q. robur*. Regarding the commercial starch, acorn starches had lower gelatinization temperatures and enthalpies, lower in vitro digestibility, lower resistance towards deformation, superior pseudoplastic behavior, and overall higher solubility and swelling power until 80 °C. This encourages the usage of acorn starches as a new food ingredient.

## 1. Introduction

Native to the Northern Hemisphere, oak trees of the *Quercus* spp. range from temperate to tropical latitudes in the Americas, Asia, Europe, and North Africa. They are evergreen, semi-deciduous, or deciduous trees that bear a small fruit called acorns [1]. There are large amounts of acorns in Portugal, and it is estimated that more than 400,000 tons are produced annually [2]. Despite being a novel food rich in resistant starch, more than half of the production is underused [3].

Native starch is formed by granules that are almost exclusively formed by amylose and amylopectin. Amylose is a linear chain of α-D-(1,4)-glucose residues, but amylopectin is highly branched since it has additional α-D-(1,6)-glucose bounds [4]. These structural differences cause amylose to have lower solubility, viscosity, gelatinization and melting temperatures, poorer thickening ability, adhesive forces, and freezing–thawing stability, but higher shear stability, a retrogradation tendency, and the ability to complex with lipids when compared to amylopectin [4]. Native starches are also a low-cost, renewable, and biodegradable resource with high availability. Still, their insolubility in cold water, fast easy aging, fast syneresis, fast retrogradation, low viscosity and shear stress resistance, and poor thermal properties and mechanical resistance limit their applicability [5]. To endow starch properties, they are usually chemically modified by the food industry through esterification, etherification, and oxidation methods to be used as food additives [6]. Oxidized starch, monostarch phosphate, distarch phosphate, phosphated distarch phosphate, acetylated distarch phosphate, acetylated starch, acetylated distarch adipate, hydroxypropyl starch, hydroxypropyl distarch phosphate, starch sodium octenyl succinate, acetylated oxidized starch, and starch aluminum octenyl succinate are the twelve authorized chemically modified starches used as food additives in the European Union [7]. However, the increasing awareness of consumers and the tendency to avoid modified starches make it necessary to produce clean-label starches [8].

High hydrostatic pressure (HP) is a non-thermal physical starch modification technology that endows starch properties from several botanical sources by using water and applying pressure levels from 100 to 600 MPa for 2 to 30 min at room temperature [9]. HP can inhibit the retrogradation of oat starch [10], increase the viscosity and obtain a highly structured profile of potato starch [11], and improve starch digestibility by decreasing the rapid and slowly digestible starch fractions and by increasing the resistant starch fraction of pea starch [12], and by increasing the swelling and solubility of corn and wheat starch [13]. HP has been gaining more attention since it does not use chemical reagents, is safer and of simple execution, and is more sustainable and environmentally friendly than the current chemical modification methods. As no chemical changes are induced, these starches do not need to be labeled as “modified starch” according to Annex I, paragraph 19 of Regulation (EC) 1333/2008 [7]. Thus, HP-modified starches can be more advantageous by attending to modern consumer demands. As HP is already used in the food industry, this study intends to use this technology to value natural resources existing in large quantities, such as acorns, to obtain food products or ingredients with high added value and great applicability, such as starch. In addition, this study also seeks to find out how the properties of these starches are comparable to commercial starch since it is familiar to the consumers and industry. Hence, this research aims to evaluate the effect of pressurization on the structure and properties of *Q. pyrenaica,* and *Q. robur* acorn starch and compare them to commercial corn starch.

## 2. Results and Discussion

### 2.1. Granular Morphology

**Figure 1:**
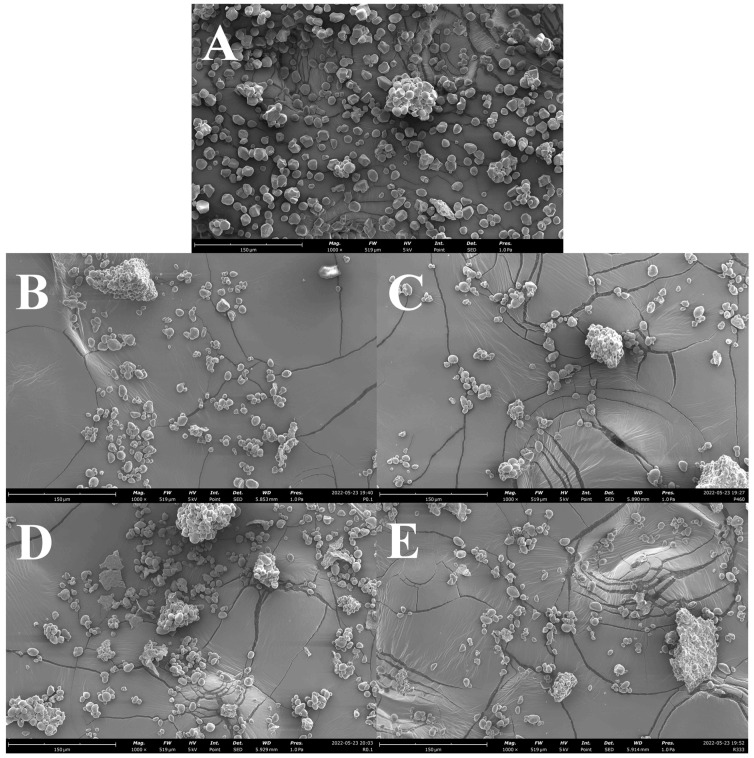
Scanning electron microscopy images at 1000× magnitude of (**A**) commercial starch; (**B**) *Q. pyrenaica* acorn starch extracted under control conditions (0.1 MPa for 20 min); (**C**) *Q. pyrenaica* acorn starch extracted under optimum conditions (460 MPa for 20 min); (**D**) *Q. robur* acorn starch extracted under control conditions (0.1 MPa for 17.4 min); (**E**) *Q. robur* acorn starch extracted under optimum conditions (333 MPa for 17.4 min).

Commercial starch granules occur in a range of shapes, as shown in Figure 1A, ranging from round, oval, and semicircular to triangular and trapezoidal–triangular, including granules with irregular shapes. Acorn starch granules were found to be round, oval, and semi-circular in shape, with some granules being triangular or trapezoidal–triangular in shape (Figure 1B–E), as reported in the literature [14,15,16,17,18,19,20,21,22]. Aside from the shape, commercial and acorn starch granules had a smooth surface without cracks and/or fissures, but a few granules had pits (Figure 1A–E) [14,17,19,20,21]. Pressurization did not affect the shapes and morphology of acorn starches of both *Q. pyrenaica* and *Q. robur* species, as previously observed for lily, chestnut, rice, and maize starches up to 500 MPa [9,23]. According to prior research, the outside layer of granules has a higher degree of order and appears to be more resistant to pressurization than the interior layer. This suggested that most changes occurred in the interior structure of the starch granule during pressurization [24]. Acorn starch granules had particles on the surface, which might be attributed to the presence of protein and/or fiber retained during extraction and sieving (Figure 1B–E) [14,16,25].

### 2.2. Granular Distribution and Particle Size

The size and shape of starch granules are related to their botanical origin and are genetically controlled. During their biosynthesis in amyloplasts or chloroplasts, the physical structures of plastids can give a certain shape to the granules and also affect the arrangement of amylose and amylopectin [26]. Hence, it is important to characterize them in terms of their distribution but also according to their size.

In the case of *Q. pyrenaica,* the commercial and acorn starches (P0.1/t20 and P460/t20) had a non-normal distribution (Figure S1). While the acorn granule distribution was wider and flatter, the commercial granule distribution was narrow and taller. This finding is supported by the span values, which were lower for commercial starch (0.9 ± 0.0 μm) compared to the acorn starches (3.7 ± 0.1 μm for P0.1/t20 and 13.0 ± 0.4 μm for P46/t20). The P0.1/t20 and P46/t20 acorn starches had a statistically higher percentage of very small (<5 μm), small (5–10 μm), and large (>25 μm) granules than commercial starch (Table 1). However, commercial starch had a percentage of medium granules (10–25 μm) significantly higher (79.1 ± 0.0%) than acorn starches (44.0 ± 0.1% for P0.1/t20 and 37.1 ± 0.2 μm for P460/t20). The commercial starch showed a uniformity dispersion value of 0.3 ± 0.0, while acorn starches P0.1/t20 and P460/t20 showed a uniformity dispersion value of 1.4 ± 0.1 and 3.4 ± 0.1, respectively. The uniformity dispersion value is a measure of the size dispersion of the starch granules. Thus, a smaller uniformity dispersion value indicates a smaller size dispersion of the starch granules and, consequently, a greater similarity between granules [27]. Hence, the commercial starch granules are more homogeneous in size and more like each other than the acorn starch granules (Figure 1).

Pressurization from P0.1/t20 to P460/t20 led to the transformation of a binomial to a trinomial distribution and flattened the distribution profile (Figure S1). The percentage of granules up to 25 µm decreased by 22% from P0.1/t20 to P460/t20 and the percentage of large granules (>25 µm) increased by 78%, which increased the span and uniformity dispersion values by 141 and 256%, respectively from P0.1/t20 to P460/t20 (*p* < 0.05) (Table 1). These results indicate that starch granule aggregation may have occurred from P0.1/t20 to P460/t20, resulting in the elongation of the granule distribution and greater heterogeneity of starch granules. The hypothesis of granular aggregation occurrence under pressurization from P0.1/t20 to P460/t20 is corroborated by the significant increases of the D_10_, D_50_, and D_90_ values by 23, 39, and 358%, respectively, indicating an increase in the maximum particle diameter below which 10, 50, and 90% of the starch granule volume exists, especially at D_90_. Indeed, there was a significant increase in the De Brouckere diameter by 174%, a sensitive measurement of the presence of large particulates in the size distribution [28]. The significant variation of the Sauter diameter, defined as the diameter of a sphere with the same volume/surface area ratio as the particle of interest [29], indicates that the mean size of the starch granule distribution increased by 37%. The increase in the Sauter diameter could have been due to the significant decrease of the specific surface area by 27% (Table 1). The increase in D values, span, and uniformity dispersion after pressurization is what was previously observed for potato, lotus, lily, pea, maize, and quinoa starch [9,23,30,31].

Regarding *Q. robur*, the commercial and acorn starches (R0.1/t17.4 and R333/t17.4) had a non-normal distribution (Figure S1). These acorn granular distributions were also significantly wider and flatter (span value of 7.2 ± 0.1 μm for R0.1/t17.4 and 6.3 ± 0.1 μm for R333/t17.4) when compared to the commercial starch. The R0.1/t17.4 and R333/t17.4 acorn starches had a higher percentage of very small, small, and large granules than the commercial starch, which had more medium granules (Table 1). Since the commercial starch had a lower uniformity dispersion value than the R0.1/t17.4 and R333/t17.4 starches (2.4 ± 0.1 and 2.0 ± 0.0, respectively), the commercial granules were more homogeneous than the acorn starch granules. Pressurization maintained the binomial distribution of the starch granules from R0.1/t17.4 to R333/t17.4, even though there was a small narrowing (Figure S1). The percentage of granules up to 25 µm increased by 6% from R0.1/t17.4 to R333/t17.4 and the percentage of large granules (>25 µm) decreased by 14%, which significantly decreased the span and uniformity dispersion values by 15 and 12%, respectively. These results indicate that starch granule disassociation may have occurred, strengthening the distribution and yielding a higher homogeneity of granules. Such a hypothesis is found within the significant decrease of the D_10_, D_50_, and D_90_ values by 5, 7, and 18%, respectively, indicating an increase in the maximum particle diameter below which 10, 50, and 90% of the starch granule volume exists. Indeed, there was a significant increase in the De Brouckere diameter by 18%. Moreover, the significant variation of the Sauter diameter indicates that the mean size of the starch granule distribution decreased by 5%. The increase in the Sauter diameter could have been due to the significant increase of the specific surface area by 5% (Table 1).

The percentage of very small, small, medium, and large granules found for *Q. pyrenaica* (P0.1/t20; 9.3 ± 0.0, 24.5 ± 0.1, 44.0 ± 0.1, and 22.2 ± 0.2%, respectively) and *Q. robur* (R0.1/t17.4; 8.2 ± 0.0, 23.8 ± 0.0, 37.9 ± 0.0, and 30.1 ± 0.1%, respectively) were comparable to those found for *Q. wutaishanica* (10.6, 34.5, 40.8, and 14.14%) [16].

### 2.3. Amylose and Amylopectin

Regarding amylose, the commercial starch had a significantly lower content when compared to the P0.1/t20 and R0.1/t17.4 starches (Table 2).

The amylose content from *Q. pyrenaica* and *Q. robur* starch increased significantly by 27% from P0.1/t20 to P460/t20 and 13% from R0.1/t17.4 to R333/t17.4, respectively. Previous authors also observed an increase in the amylose starch content after pressurization for litchi, maize, mango, potato, sweet potato, buckwheat, and sorghum starches [9,32,33,34]. The amylose contents of *Q. pyrenaica* (P0.1/t20; 53.2 ± 0.5%) and *Q. robur* starches (R0.1/17.4; 58.2 ± 0.1%) were similar to those reported for *Q. rotundifolia* (53.7–54.5%) and *Q. suber* (57.9–59.4%) starches, respectively [35]. However, they were higher than those reported for *Q. ilex* (25.8%) [36], *Q. palustris* (31.4%) [18], *Q. serrata* (27.1%) [37], *Q. suber* (24.4%) [38], *Q. calliprinos* (29.2%) [39], *Q. suber* (34.4%) [36], *Q. acutissima* (30.6%) [15], *Q. ilex* (31%) [40], and *Q. wutaishanica* (31.4%) [16], *Q. ilex* (39.0%) [41], *Q. pubescens* (19.5%) [36], *Q. rotundifolia* (41.7%) [42], *Q. leucotrichophora* (15.6%) [14], *Q. brantii* (~18%) [21], *Q. coccifera* (36%) [40], and *Q. suber* (48.9%) [42] starches. The mechanical forces created during pressurization may have led to the cleavage of covalent bonds along the polymeric chains. Since amylopectin is more prone to degradation than amylose, the higher amylose content was associated with the cleavage of α-D-(1,6)-glucose bounds [43]. Indeed, the amylopectin contents decrease significantly after pressurization by 28% from P0.1/t20 to P460/t20 for *Q. pyrenaica* and 43% from R0.1/t17.4 to R333/t17.4 for *Q. robur*, respectively (Table 2).

### 2.4. Damaged Starch

For *Q. pyrenaica*, both P460/t20 and commercial starches had a significantly higher content when compared to the P0.1/t20 starch (Table 2). Pressurization from P0.1/t20 to P460/t20 increased significantly the damaged starch content by 584%, which may have been due to the rearrangement of amylose and amylopectin, leaving the chains more susceptible to enzymatic action and/or amylopectin degradation into amylose [30]. Previous authors also observed an increase in the damaged starch content from quinoa, lentils, potato, sweet potato, and maize starches under pressurized conditions [30,31,33,44,45]. For *Q. robur*, both commercial and R333/t20 starches had similar contents when compared to the R0.1/t17.4 starch, but the damaged starch content increased by 91% from R0.1/t17.4 to R333/t17.4. These results show that *Q. pyrenaica* starch is more susceptible to pressurization than *Q. robur* starch. For *Q. pyrenaica*, it is thought that a few short chains of amylopectin may be in the form of a double helix, causing water molecules to enter the crystalline zones of the starch granules more easily during pressurization and thus destroying the outer layer of the starch granule structure. Regarding *Q. robur*, a greater part of these chains may be associated with a double helix, which makes the penetration of water molecules difficult, making it necessary to apply higher pressure levels to destroy the crystalline structures [33]. However, future analyses of the distribution of different amylopectin sub-chains should be performed to obtain more detailed information and thus better explain the observed results.

### 2.5. Fourier Transform Infrared Spectroscopy

Starches showed similar FTIR spectra, but the spectra of pressurized acorn starches (P460/t20 and R333/t174) were more similar to the spectrum of the commercial starch than the corresponding native acorn starches (P0.1/t20 and R0.1/t174) (Figure S2).

Regarding the degree of short-range molecular order of *Q. pyrenaica*, both commercial and P460/t20 starches had similar but significantly lower 1047/1022 ratios when compared to P0.1/t20 starch (Table S1). Furthermore, the 1047/1022 ratio decreased significantly by 2% after pressurization from P0.1/t20 to P460/t20, which indicates a loss of the ordered structure. For *Q. robur*, the commercial starch had significantly lower 1047/1022 ratios when compared to both R0.1/t17.4 and R333/t17.4 starches. The 1047/1022 ratio also decreased significantly after pressurization R0.1/t17.4 and R333/t17.4 but by 10%, showing a higher loss of ordered structure (Table S1). The 1047/1022 ratios of *Q. pyrenaica* starch (P0.1/t20; 1.079 ± 0.001) were more similar to those previously reported for *Q. wutaishanica* (1.049) [16] and *Q. variabilis* starches (1.082) [17] than that of *Q. robur* starch (R0.1/17.4; 1.194 ± 0.007) found in the present paper.

Concerning the internal changes in the double helix degree of *Q. pyrenaica*, the commercial starch had statistically lower 995/1022 ratios than the P0.1/t20 and P460/t20 starches (Table S1). The 995/1022 ratio decreased significantly by 4% from P0.1/t20 to P460/t20, indicating a weakening hydrogen bond between amylopectin chains of double helix structures [10,46]. For *Q. robur*, the commercial starch also had statistically lower 995/1022 ratios than the R0.1/t17.4 to R333/t17.4 starches. The 995/1022 ratio also decreased significantly after pressurization from R0.1/t17.4 to R333/t17.4 but by 18%, which shows higher internal changes of the double helix. These results agree with each other since the loss of an ordered structure will also imply a loss of the double helix degree.

### 2.6. X-ray Diffraction

**Figure 2:**
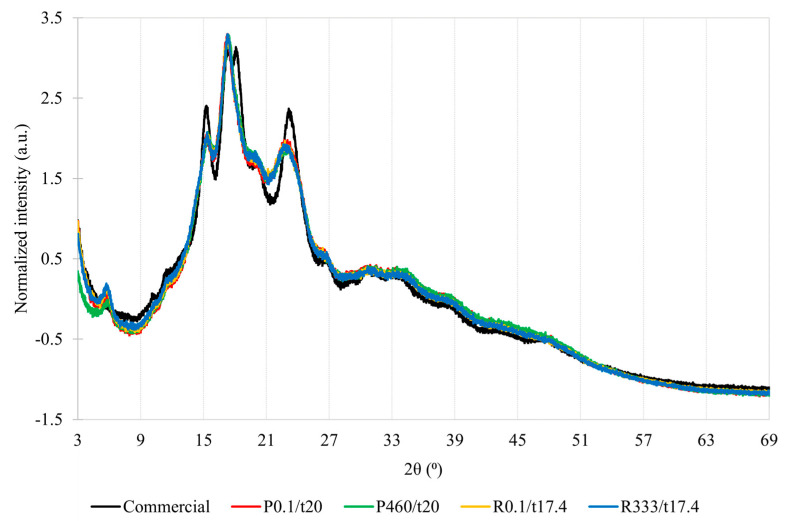
X-ray diffraction pattern of the commercial starch (black line); *Q. pyrenaica* acorn starch extracted under control conditions (red line; 0.1 MPa for 20 min—P0.1/t20); *Q. pyrenaica* acorn starch extracted under optimum conditions (green line; 460 MPa for 20 min—P460/t20); *Q. robur* acorn starch extracted under control conditions (yellow line; 0.1 MPa for 17.4 min—R0.1/t17.4); *Q. robur* acorn starch extracted under optimum conditions (blue line; 333 MPa for 17.4 min—R333/t17.4).

The crystalline structures of the amylopectin double chains can have different arrangements and can be mainly categorized as type-A, B, or C, according to the X-ray diffraction pattern [47]. The unit cell of type-A crystals is formed by seven double helices in a staggered monoclinic lattice with eight water molecules. In comparison, type-B consists of six double helices enclosing a large void that can harbor up to 36 water molecules. Type-C is believed to be a combination of type-A and type-B. The commercial starch had an unresolved doublet at 2θ = 17.3° and 18.1° and two peaks at 2θ = 15.3° and 23.2°, which is typical of the type-A diffraction pattern. Acorn starches showed a typical type-C diffraction pattern with four single diffraction peaks at 2θ = 5.7, 15.3, 17.3, and 23.0° with an additional faint peak at 2θ = 20.1° possibly due to starch–lipid complexes (Figure 2).

This diffraction pattern, observed for the current *Q. pyrenaica* and *Q. robur* starches, was also found for *Q. rotundifolia* [48], *Q. suber* [48], *Q. fabri* [22], *Q. brantii* [20], and *Q. wutaishanica* [16], but a type-A diffraction pattern was found for *Q. ilex* [49], *Q. glandulifera* [50], *Q. acutissima* [15], *Q. palustris* [18], *Q. suber* [51], and *Q. variabilis* [17]. According to the literature, the type-A and type-C patterns tend to shift towards type-B after pressurization whilst type-B is usually kept. Since the diffraction peaks and pattern were preserved after pressurization, it is thought that the compressive effect of pressure might have been felt on the amorphous regions and/or the pressure level was not sufficient to alter the crystalline structures of the amylopectin double chains due to the high amylose content [47,52].

Regarding relative crystallinity, commercial starch showed a significantly higher value than acorn starches, but pressurization had no impact on acorn starch from *Q. pyrenaica* and *Q. robur* (Table S1). The relative crystallinity values of *Q. pyrenaica* (P0.1/t20; 29.3 ± 1.3%) and *Q. robur* (R0.1/17.4; 22.4 ± 1.6%) starches were following the *Q. wutaishanica* (24.3%) [16], *Q. glandulifera* (23.5%) [50], and *Q. palustris* (22.3%) [18] starches, but were inferior to those found for *Q. variabilis* (30.6%) [17], *Q. rotundifolia* (43.1–46.6%) and *Q. suber* (43.1–44.0%) [48], *Q. fabri* (48.2%) [22], and *Q. brantii* (47.8%) [20]. These results may seem contradictory to those verified in the FTIR analysis; however, it is necessary to keep in mind that the crystallinity calculation in the X-ray analysis is relative since it is performed based on the integration of areas of crystalline and amorphous domains from the diffractogram. Therefore, this determination is thought to be less sensitive to minor variations than the one that is inferred based on the FTIR analysis.

### 2.7. Solubility and Swelling Power

The solubility values of the different starches increased with the corresponding swelling power values (r^2^ < 0.96, *p* > 0.05), showing that solubilization and granular swelling occurred simultaneously, as previously reported for *Q. rotundifolia* and *Q. suber* [35]. Such can be justified by considering that a fraction of amylose can be entangled within the amylopectin, while the remaining fraction is free [14]. Concerning solubility, no solubilization was seen at 50 °C but solubilization increased significantly from 60 to 100 °C (Table 3).

When heated under enough temperature, the crystalline regions of starch granules are broken and hydrogen bonds are formed between the water molecules and the free hydroxyl groups of amylose and amylopectin. This enhances the water absorption capacity and solubility, the latter due to amylose leaching [22]. For *Q. pyrenaica*, the solubility values of the P0.1/t20 and P460/t20 starches measured until 80 °C were higher than the commercial starch (Table 3). However, the solubility values of the P0.1/t20 and P460/t20 starches measured at 90–100 °C were lower than the commercial starch. The higher solubility values of acorn starch at lower temperatures in relation to the commercial starch encourages the usage of acorn starch as an food additive in fermented yoghurt and milk products [49]. Pressurization from P0.1/t20 to P460/t20 led to an overall significant decrease in the solubility values measured at 60–100 °C. Regarding *Q. robur*, solubility values at 60–80 °C of the R0.1/t17.4 and P333/t17.4 starches were statistically inferior and/or similar to the commercial starch (Table 3). The commercial starch had significantly higher solubility at 90–100 °C than the R0.1/t17.4 and P333/t17.4 starches. Pressurization from R0.1/t17.4 to P333/t17.4 did not change the solubility values until 70 °C, but they decreased significantly at 80–100 °C. It is considered that the reduction in solubility may be due to a greater entanglement of amylose caused by the increase in its content (Table 2).

In the matter of swelling power, no swelling power was seen at 50 °C. However, the swelling power increased significantly from 60 to 100 °C (Table 3). Concerning *Q. pyrenaica*, the swelling power of the commercial starch measured until 80 °C was significantly lower than those of the P0.1/t20 and P460/t20 starches. However, the commercial starch had significantly higher swelling power values at 90–100 °C than the P0.1/t20 and P460/t20 starches. Pressurization from P0.1/t20 to P460/t20 did not change the swelling power values until 60 °C, but a significant reduction was observed from 70 to 100 °C. Previous studies in the literature also report a decrease in both solubility and swelling power for lentils, wheat, quinoa, maize, potato, sweet potato, rice, kidney bean, corn, and waxy corn starch [27,33,44,53,54,55]. For *Q. robur*, the swelling power values of the commercial starch were generally lower than R0.1/t17.4 and R333/t17.4 starches up to 80 °C. However, the commercial starch showed higher values than both the R0.1/t17.4 and R333/t17.4 acorn starches at 100 °C. However, pressurization from R0.1/t17.4 to R333/t17.4 did not significantly alter the swelling power values up to 90 °C. However, there was a significant reduction at 100 °C.

Swelling power is a measurement of the granule water-holding capacity, i.e., water absorption and retention capacity of starch granules [56]. During pressurization, the amylose and amylopectin chains could have been altered, thus limiting the swelling power [55]. So, in other words, it is thought that the observed reduction in swelling power may have been caused by the decrease in solubility and/or cleavage of the amylopectin chains (Table 2). Granular disintegration (as evidenced by increased contents of damaged starch) could also inhibit amylose solubilization and lead to a lower solubility and swelling power [33]. The observed limited swelling power values from 60 to 100 °C could have been due to strong interactions between starch chains due to the high amylose content and/or the presence of amylose–lipid complexes [14,35] (Figure 2 and Table S1).

The solubility and swelling power values of *Q. pyrenaica* (P0.1/t20; 2.36 ± 0.18–17.82 ± 0.18% and 6.40 ± 0.10–16.83 ± 0.03 g/g, respectively) and *Q. robur* (R0.1/17.4; 1.46 ± 0.19–19.71 ± 0.14% and 6.53 ± 0.09–16.75 ± 0.60 g/g, respectively) starches diverged from those of *Q. brantii* (9.7% and 24.5 g/g, respectively) [20], *Q. fabri* (0.26–4.87% and 0.024–0.14 g/g, respectively) [22], *Q. brantii* (60–65% and 0.26–0.27 g/g, respectively) [21], *Q. rotundifolia* (9.1 g/g) [42], *Q. suber* (9.0 g/g) [42], and *Q. ilex* (0.2–14.0% and 8.95–13 g/g, respectively) [41].

### 2.8. Differential Scanning Calorimetry

**Figure 3:**
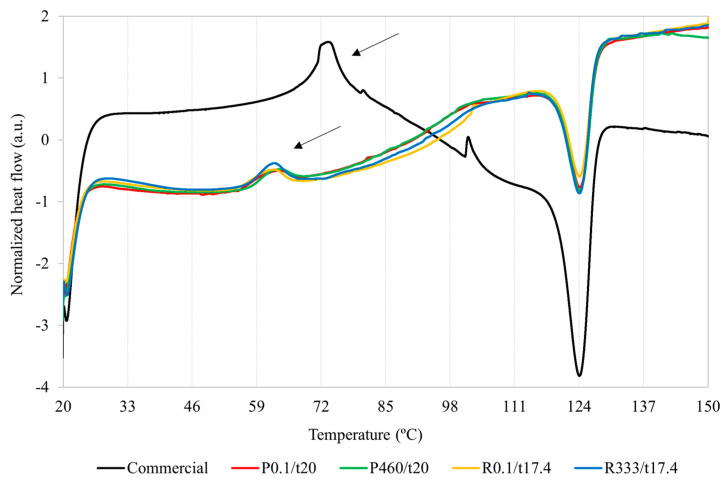
DSC thermograms of the commercial starch (black line); *Q. pyrenaica* acorn starch extracted under control conditions (red line; 0.1 MPa for 20 min—P0.1/t20); *Q. pyrenaica* acorn starch extracted under optimum conditions (green line; 460 MPa for 20 min—P460/t20); *Q. robur* acorn starch extracted under control conditions (yellow line; 0.1 MPa for 17.4 min—R0.1/t17.4); *Q. robur* acorn starch extracted under optimum conditions (blue line; 333 MPa for 17.4 min—R333/t17.4). The black arrows point to the endothermic phenomena corresponding to starches’ gelatinization.

It was verified that starch gelatinization is an endothermic phenomenon characterized by a positive increase in the enthalpies of the system (indicated by the black arrows in Figure 3).

Regarding the gelatinization temperatures, the commercial starch showed significantly higher T_o_, T_p_, and T_c_ temperatures than the acorn starches (Table S2). On the one hand, this shows that commercial starch requires higher temperatures to trigger gelatinization than acorn starches. Thus, the hydrogen bonds between amylose and/or amylopectin appear to be stronger in commercial starch than in acorn starches. These results agree with those in Table 2 since gelatinization (i.e., amylose leaching and solubilization) can occur only when the temperature used is equal to or higher than T_o_. Furthermore, the solubility of the commercial starch at 60 °C was lower than the acorn starches due to differences in the T_o_ temperatures.

Pressurization did not affect the T_o_, T_p_, and T_c_ temperatures of both acorn starch species, as previously reported for chestnut flour [25]. The T_o_, T_p_, and T_c_ values found for *Q. pyrenaica* (P0.1/t205; 5.4 ± 0.3, 62.9 ± 0.4, and 68.6 ± 1.3 °C, respectively) and *Q. robur* (R0.1/17.4; 55.0 ± 0.4, 62.3 ± 0.2, and 66.8 ± 0.3 °C) starches were generally lower to those of *Q. wutaishanica* (60.1, 70.5, and 79.3 °C, respectively) [16], *Q. fabri* (60.5, 63.8, and 70.2 °C, respectively) [22], *Q. palustris* (65.0, 73.7, and (-) °C, respectively) [18], *Q. brantii* (60.5, 63.8, and 70.2 °C, respectively) [20], *Q. acutissima* (60.1, 69.5, and 80.6 °C, respectively) [15], *Q. glandulifera* (60.8, 66.5, and 73.8 °C, respectively) [50], *Q. ilex* (61.0–75.1, 17.8–88.1, and 98.0–120.0 °C, respectively) [41], *Q. rotundifolia* (58.7–60.9, 66.7–65.7, and 74.0–74.7 °C, respectively) [35], and *Q. suber* (58.4–58.6, 64.1–64.8, and 71.4–73.3 °C, respectively) [35] starches.

Since the gelatinization temperatures were not affected after pressurization, this reinforces the hypothesis that the decrease in solubility and swelling power of starches after pressurization could have been due to the formation of lipid–amylose complexes, as well as the scenario that the observed limited swelling power values were attributed due to strong interactions between amylose and/or amylopectin and/or the presences of amylose–lipid complexes [14,35].

Regarding gelatinization enthalpies, the commercial starch also showed significantly higher gelatinization enthalpies than the acorn starches (Table S2). This shows that commercial starch requires a higher energy input to disrupt the hydrogen intra-helix bonds of the crystalline regions (i.e., initiate and complete the gelatinization process) when compared to the acorn starches since the commercial starch had significantly higher gelatinization temperatures. Yet, pressurization did change the gelatinization enthalpies of acorn starches, as reported for chestnut flour [25]. The gelatinization enthalpies found for the *Q. pyrenaica* (P0.1/t20; 12.7 ± 1.5 J/g) and *Q. robur* (R0.1/17.4; 11.9 ± 1.3 J/g) starches are in accordance to those found for the *Q. brantii* (14.9 J/g) [20], *Q. acutissima* (9.7 J/g) [15], and *Q. fabri* (10.9 J/g) [22] acorn starches, but diverge from the *Q. palustris* (20.8 J/g) [18], *Q. suber* (4.2–4.3 J/g) [35], *Q. wutaishanica* (4.3 J/g) [16], *Q. glandulifera* (4.3 J/g) [50], and *Q. rotundifolia* (4.2–4.3 J/g) [35] starches.

It is thought that the commercial starch would have shown a greater relative crystallinity than the acorn starches since it had a lower amylose/amylopectin ratio (Table 2 and Table S1). In other words, a lower amylose content makes it possible for amylopectin chains to form more crystalline domains. Since these interactions between chains involve the formation of hydrogen bonds, the gelatinization temperatures will be higher and, consequently, more energy will have to be supplied to break the hydrogen bonds (higher enthalpies of gelatinization) (Table S2).

### 2.9. In Vitro Digestibility

Concerning *Q. pyrenaica*, both P460/t20 and commercial starches were shown to have statistically more digestible starch than P0.1/t20 starch (Table 4). However, the P460/t20 starch had significantly more RDS, but less SDS and TDS than the commercial starch. Furthermore, the RDS, SDS, and TDS contents increased significantly by 176, 52, and 64%, respectively from P0.1/t20 to P460/t20, while the RS content decreased by 47%. In other words, the increase of RDS and SDS contents might have occurred by the transformation of RS. The decrease of the RS contents may have resulted from structural modifications and rupture of starch molecules, mainly the cleavage of α-(1,6)-glycosidic bonds of amylopectin (Table 2).

These results are similar to those for wheat, potato, sweet potato, rice, kidney bean, corn, waxy wheat, waxy rice, and waxy corn starch [53,55,57,58]. Regarding *Q. robur*, there were no significant differences in the RS contents from R0.1/t17.4 to R333/t17.4, which were statistically higher than the RDS and SDS contents of the commercial starch. The pressure level applied may have been insufficient to destroy the starch granule structure [58]. No differences were also found in the RDS and TDS contents from R0.1/t17.4 to R333/t17.4, which were statistically lower than the RDS and TDS contents of the commercial starch. The commercial starch had a significantly higher SDS content than both *Q. robur* starches, but the SDS contents decreased by 46% from R0.1/t17.4 to R333/t17.4.

Pressurization may have stabilized amylopectin and double-amylose molecules by increasing the content of ^4^C_1_ chair conformations and/or strengthening the Van der Waals and electrostatic forces of adjacent chains, which may have led to the reduction of interchain distances and surface area of both the amylopectin and double-amylose molecules [59]. These more compacted molecules can then be related to a decreased susceptibility of starch to amylolytic enzymes. These results indicate that the different starch fractions from *Q. pyrenaica* and *Q. robur* are affected differently when pressurized to the corresponding optimum extraction conditions. The RDS, SDS, and RS contents found for *Q. pyrenaica* (P0.1/t20; 7.9 ± 0.5, 18.2 ± 0.4, and 40.5 ± 1.4%, respectively) and *Q. robur* (R0./17.4; 12.3 ± 0.8, 38.1 ± 1.9, and 33.9 ± 0.1%, respectively) starches diverge from those of *Q. wutaishanica* (17.0, 22.4, and 60.6%, respectively) [16] and *Q. variabilis* starches (17.2, 23.8, and 59.0%, respectively) [17].

Concerning the total starch, no differences were found in the content between commercial and P460/t20 starches, nor between the P0.1/t20 and P460/t20 starches from *Q. pyrenaica* (Table 3). For *Q. robur*, the commercial starch presented a higher total content than the R0.1/t17.4 and R333/t17.4 starches. It is believed that such differences may be due to the presence of small contents of protein and/or fiber that may have been sieved along with the starch granules during extraction.

### 2.10. Steady Flow Behavior

The consistency coefficient and flow behavior index for all starches are summarized in Table 5. The consistency coefficient measures the starch fullness and can be seen as a viscosity criterion [54]. For *Q. pyrenaica*, the commercial and P460/t20 starches had similar consistency coefficient values concerning the P0.1/t20 starch. Pressurization from P0.1/t20 to P460/t20 significantly decreases the consistency coefficient by 34%. The same effect was observed for mango, maize, and quinoa starches [27,32]. This indicates a decrease in flow and shear stress resistance, which may have been due to the cleavage of α-(1,6)-glycosidic bounds (Table 2).

For *Q. robur*, both R0.1/t17.4 and R333/t17.4 starches had significantly higher consistency values than the commercial starch, indicating that *Q. robur* acorn starches have superior resistance to flow and shear stress than the commercial starch. However, pressurization did not affect the consistency coefficient from R0.1/t17.4 to R333/t17.4. The consistency coefficient values of *Q. pyrenaica* (P0.1/t20; 0.035 ± 0.001 Pa.s^n^) and *Q. robur* (R0.1/17.4; 0.036 ± 0.004 Pa.s^n^) had a similar order of magnitude to that found for *Q. brantii* (0.011 Pa.s^n^) [60].

Concerning the index of flow behavior, all analyzed starches had values lower than 1, indicating that starches were pseudoplastic fluids (Table 5). For *Q. pyrenaica*, the commercial and P460/t20 starches showed a similar index of flow behavior when compared to P0.1/t20 starch. Pressurization from P0.1/t20 to P460/t20 led to a significant increase in the flow behavior index by 10%. The same effect was observed for mango, maize, and quinoa starches [27,32]. This indicates an increase in fluidity (associated with the decrease of the consistency coefficient), thus weakening the pseudoplastic behavior.

Since *Q. pyrenaica* starch showed a much greater increase in amylose after pressurization than *Q. robur* starch (Table 2), the α-D-(1,4)-glucose chains of P460/t20 starch could not have the same anisotropic capacity as P0.1/t20 starch, decreasing the index values of flow behavior. In the case of *Q. robur*, both R0.1/t17.4 and R333/t17.4 starches had a significantly lower index of flow behavior than the commercial starch, indicating that *Q. robur* acorn starches have a superior pseudoplastic behavior. Pressurization had no effect on the index of flow behavior from R0.1/t17.4 to R333/t17.4. The index of the flow behavior of *Q. pyrenaica* (P0.1/t20; 0.782 ± 0.003) and *Q. robur* (R0.1/17.4; 0.780 ± 0.023) was in agreement with that found for *Q. brantii* (0.780) [60].

### 2.11. Dynamic Oscillation

Figure S3 shows the effect of pressurization on the complex modulus and complex viscosity of all starches analyzed. The complex viscosity measures the total resistance to the angular frequency [61]. The complex viscosity decreased linearly with angular frequency. Still, significant differences were found among the so-called *H* values, which corresponds to the linearization slopes for each of the starches. These values show how the complex viscosity decreases with frequency among the different starches (Table 5). Regarding *Q. pyrenaica*, the commercial starch had a lower *H* value than the P0.1/t20 and P420/t20 starches. Pressurization from P0.1/t20 to P420/t20 significantly increased the *H* values, showing that the complex viscosity of P420/t20 starch decreases more sharply with increasing frequency than the P0.1/t20 starch. This indicates that pressure treatment shifted the behavior from a viscoelastic solid to a viscoelastic fluid [9]. It is thought that this result may be related to the higher amylose increase and reduced amylopectin content since amylose has a linear structure while amylopectin is highly branched (Table 2). For *Q. robur*, the commercial starch had a lower *H* value than the R0.1/t17.4 and P333/t17.4 starches, but pressurization did not affect the *H* values. The *H* values of *Q. pyrenaica* (P0.1/t20; −0.927 ± 0.005) and *Q. robur* (R0.1/17.4; −0.909 ± 0.008) were similar to *Q. brantii* (−0.94) [62].

The complex modulus measures the overall resistance of materials to deformation [61]. According to Figure S3, the complex modulus of the analyzed starches increased with frequency, but significant differences were found in the complex index between starches (Table 5). For *Q. pyrenaica*, the complex index of the commercial starch was significantly lower than that presented by the P0.1/t20 and P420/t20 starches. However, pressurizing from P0.1/t20 to P420/t20 led to a significant increase in the complex index by 23%. This represents an increase in the dependence of the complex modulus with frequency, which, in other words, translates to a decrease in resistance towards deformation. For *Q. robur*, the complex index of the commercial starch was also significantly lower than that presented by the R0.1/t17.4 and P333/t17.4 starches. Pressurizing from R0.1/t17.4 to P333/t17.4 significantly increases the complex index by 8%. These results are lower than those found for quinoa [31]. It is thought that, as pressurization led to a greater increase in amylose content in *Q. pyrenaica* than in *Q. robur*, it is considered that amylose retrogradation may have been facilitated more in *Q. pyrenaica* than in *Q. robur*, leading to a distinct increase in the complex index (Table 2).

The complex modulus is the result of the contribution of the elastic (*G*′) and viscous (*G*″) components of the materials. The former is the elastic modulus and measures the material’s ability to store energy, whilst the latter is the dissipation module and measures the ability to dissipate energy [61]. All starches displayed a predominantly solid-like behavior in the studied frequency range of 0.1–10 Hz since that *G*′ > *G*″, with no crossover (Figure S4). Both moduli increased with increasing frequency, as shown by the index values, but the increment of *G*″ was higher than that of *G*′. This indicates a slow development in the mechanical rigidity of the starch gels, as previously observed for chestnuts [25].

Regarding the elastic moduli, they behaved as the complex moduli counterparts (Table 5). In the case of *Q. pyrenaica*, pressurization from P0.1/t20 to P420/t20 significantly increases the elastic index by 21%. For *Q. robur*, pressurizing from R0.1/t17.4 to P333/t17.4 also significantly increases the elastic index by 8%. This indicates that the solid-like behavior of acorn starches decreased after pressurization, i.e., it was transformed from solid-like to a more liquid-like behavior [9]. Hence, the decrease in the overall resistance of the acorn starches to deformation can be explained by the loss of the starch’s ability to store energy. The elastic index found for *Q. pyrenaica* (P0.1/t20; 0.071 ± 0.001) and *Q. robur* (R0.1/17.4; 0.091 ± 0.000) was higher than that found for *Q. brantii* (0.05) [62].

Concerning the viscous modulus, it behaved differently than the elastic moduli (Table 5). In the case of *Q. pyrenaica*, the viscous index value of the commercial starch was similar to the one presented by P0.1/t20. However, pressurizing from P0.1/t20 to P420/t20 led to a significant and sharper increase of the viscous index by 47%. For *Q. robur*, the viscous index value of the commercial starch was also similar to the one presented by R0.1/t17.4. Pressurizing from R0.1/t17.4 to P333/t17.4 also led to a significant and sharper increase of the index by 18%. This increase represents an increase in the dependence of the viscous modulus with frequency, which translates into a higher ability to dissipate energy. The viscous index of *Q. pyrenaica* (P0.1/t20; 0.086 ± 0.008) and *Q. robur* (R0.1/17.4; 0.097 ± 0.003) was much lower than that found for *Q. brantii* (0.45) [62].

## 3. Conclusions

Pressurized starch extraction from *Q. pyrenaica* and *Q. robur* acorns under optimal conditions led to significant structural and property changes, depending on the species. Pressure affected the span distribution and uniformity of acorn starch granules, but the granular morphology remained unaffected. Pressurization increased the amylose/amylopectin ratios and damaged starch contents, particularly in *Q. pyrenaica*. However, relative crystallinity, polymorphism, gelatinization temperatures, and enthalpies remained unchanged. The properties of acorn starches were affected by pressurization, with decreased solubility and swelling power due to amylopectin depolymerization and increased complex resistance to deformation. Pressurization increased in vitro digestibility and decreased pseudoplastic behavior in *Q. pyrenaica* starch, while no differences were observed in *Q. robur*. Acorn starches may be more advantageous than commercial corn starch due to lower gelatinization temperatures, enthalpies, in vitro digestibility, superior pseudoplastic behavior, and lower resistance to deformation, which encourages acorn starch to be used as a food ingredient or additive food product, thus valorizing acorn starches.

## 4. Materials and Methods

### 4.1. Acorn Sampling and Commercial Corn Starch

On 22 November 2018, acorns of the *Q. pyrenaica* and *Q. robur* species were harvested in Parque Nacional da Peneda-Gerês, Portugal, in Assento, Terras de Bouro, and Braga. The *Q. robur* oaks were located at Parque Cerdeira (41°45′46.0″ N; 8°11′24.2″ W) and the *Q. pyrenaica* oaks were found next to Rio Homem (41°45′49.7″ N; 8°11′55.9″ W). Acorns were hand collected from the entire area of ground covered by the canopy of various oak trees according to their quality (i.e., absence of putrefaction and no mechanical damage and/or spoilage by larvae were defined as quality controls). Acorns were brought to the laboratory facilities the same day in thermal plastic bags, washed with tap water to remove soil and foliage, cleaned with a fabric cloth, and stored at −20 °C until further use.

A commercial corn starch (Hacendado^®^, Zamora, Spain) was purchased from a local supermarket.

### 4.2. Acorn Starch Extraction

The acorn shells were removed by hand with a regular kitchen knife, and the cotyledons were grounded using a regular food processor, screened using 1 mm and 500 µm mesh sieves, and finally stored at −20 °C until further usage.

An 8% (*w*/*v*) acorn flour suspension was prepared with deionized water in low-permeability polyamide-polyethylene bags (IdeiaPack—Comércio de Embalagens, Lda, Viseu, Portugal). Bags were manually heat sealed with minimum air and placed inside the high hydrostatic pressure vessel with an inner diameter of 200 mm and 2000 mm in length (Hiperbaric 55, Hiperbaric, Burgos, Spain). The industrial equipment had a maximum operating pressure of 600 MPa and was connected to a refrigeration unit (RMA KH 40 LT, Ferroli, San Bonifacio, Italy) to control the temperature of the input water used as a pressurizing fluid. Starch extraction conditions were optimized using different pressure levels (0.1, 250, and 500 MPa) and extraction times (5, 12.5, and 30 min) by response surface methodology. The compression rate used was 250 MPa/min and the decompression was instantaneous. Suspensions were centrifuged at 3000× *g* for 20 min, and the pellets were screened using 180 and 40 μm diameter mesh sieves, thoroughly washed with water, and left to stand at 4 °C overnight. The starchy material was collected and dried at 45 °C in a ventilated oven-drying chamber until constant weight.

The optimum starch extraction condition from *Q. pyrenaica* acorns was 460 MPa during 20 min and from *Q. robur* acorns was 333 MPa during 17.4 min (Table 6). Control extraction was performed at 0.1 MPa for 20 min for *Q. pyrenaica* and at 0.1 MPa for 17.4 min for *Q. robur*. Extractions were performed in triplicate.

### 4.3. Structural Characterization

#### 4.3.1. Scanning Electron Microscopy

Morphology was examined using a Phenom XL G2 (Thermo Fisher Scientific, Waltham, MA, USA) scanning electron microscope (SEM). Samples were placed on top of the observation pins covered with double-sided adhesive carbon tape (NEM tape; Nisshin, Japan). Afterwards, the samples were coated with gold/palladium on a sputter coater (Polaron, Germany) and visualized. SEM analyses were performed with the equipment operated at a high vacuum and an accelerating voltage of 20 kV using the secondary electron detector. Morphology was accessed according to the literature [63].

#### 4.3.2. Particle Size

Measurements were performed in triplicate using a Mastersizer Hydro EV 3000 (Malvern Instruments Ltd., Worcestershire, UK) by laser diffraction with a constant paddle rotation of 2100 rpm. The absorption index used was 0.10 and the refractive indices of water and starch were 1.33 and 1.53, respectively. The D_10_, D_50_, and D_90_ represented the particle sizes at which 10, 50, and 90% of all the particles by volume were smaller, respectively, and the Sauter and De Brouckere mean diameters (D_3,2_ and D_4,3_, respectively) were recorded. Uniformity dispersion and span values were also registered. Granules were classified as large (>25 µm), medium (10–25 µm), small (5–10 µm), and very small (<5 µm) [26].

#### 4.3.3. Damaged Starch, Amylose, and Amylopectin

Damaged starch was quantified in duplicate using a commercial kit from Megazyme, Wicklow, Ireland (REF: K-SDAM) based on the hydration of damaged granules and hydrolysis into maltosaccharides and α-limit dextrins with fungal α-amylase. Results were expressed in percentage (grams of damaged starch per 100 g of starchy material) on a dry basis.

Amylose was quantified in duplicate using a commercial kit from Megazyme International, Wicklow, Ireland (REF: K-AMYL) based on amylopectin precipitation by concanavalin A lectin. Amylopectin was computed by the difference between the total starch and the amylose content. Results were expressed in percentage (grams of amylose or amylopectin per 100 g of starchy material) on a dry basis.

#### 4.3.4. Fourier Transform Infrared Spectroscopy

Measurements were performed in triplicate in the mid-infrared region with a resolution of 4 cm^−1^ using a Spectrum 100 FT-IR Spectrometer (PerkinElmer, MA, USA). The measurement was conducted at room temperature, and spectra were recorded in the 400–4000 cm^−1^ range at 64 scans.

#### 4.3.5. X-ray Diffraction

Diffractograms were obtained in duplicate using a MiniFlex 600 diffractometer (Rigaku, Tokyo, Japan) with Cu-Kα radiation using a voltage of 40 kV and a current of 15 mA. Starches were scanned from 3 to 70° at a rate of 3°/min at a 0.01° step. Relative crystallinity (RC) was computed according to Equation (1), where *C_a_* is the crystalline area and *A_a_* is the amorphous area.

(1) $$\mathrm{RC}\;\left(\%\right)=\left(\frac{C_{a}}{C_{a}\;+\;A_{a}}\right)\;\times \;100$$

### 4.4. Property Characterization

#### 4.4.1. Solubility and Swelling Power

Solubility (S) and swelling power (SP) were measured in duplicate according to [35]. A starch suspension in water (1%, *w*/*v*) was incubated in a water bath for 30 min from 50 to 100 °C. Suspensions were then cooled and centrifuged at 4100× *g* for 30 min. The supernatants were freeze dried using a vertical freeze dryer BK-FD12P (Biobase, Shandong, China) and weighed, whilst gels were weighed as is. Solubility and swelling power were computed according to Equations (2) and (3), respectively, where *W_LS_* is the weight of the lyophilized supernatant (g), *W_S_* is the starch weight in dry basis (g), and *W_G_* is the gel weight (g). Solubility and swelling power results were expressed in percentage (%; gram of solubilized starch per 100 g of starchy material on a dry basis) and gram of gel per g of non-solubilized starchy material on a dry basis (g/g SM), respectively.

(2) $$S\;(\%)=\left(\frac{W_{LS}}{W_{S}}\right)\;\times \;100$$

(3) $$\mathrm{SP}\;(g/g)=\frac{W_{G}}{W_{s}\;\times \;\left(1\;-\frac{S}{100}\right)}$$

#### 4.4.2. Differential Scanning Calorimetry

Thermal characteristics were determined in triplicate using Diamond differential scanning calorimetry equipment (PerkinElmer, Shelton, CT, USA) [35]. The equipment was calibrated with indium and lead for temperature and heat capacity calibration. A mass of 3 mg of starch was weighed into a stainless-steal pan (REF: 03190218, PerkinElmer) and 11 µL of deionized water was added. After being hermetically sealed and left to equilibrate for 2 h at room temperature, the container was heated to 150 °C at 10 °C/min. An empty stainless-steal pan was used as a reference and the flow rate of the nitrogen was 40 mL/min. The onset (T_o_; °C), peak (T_p_; °C), and conclusion (T_c_; °C) temperatures, and gelatinization enthalpy (∆H) were obtained using the Pyris software (v13.4.0).

#### 4.4.3. In Vitro Digestibility

The rapidly digestible starch (RDS), slowly digestible starch (SDS), total digestible starch (TDS), and resistant starch (RS) fractions were quantified in duplicate using a commercial kit from Megazyme, Wicklow, Ireland (REF: K-DSTRS). Total starch content was defined as the sum of the total digestible (TDS) and resistant starch (RS) fractions. Results were expressed in percentages (grams per 100 g of starchy material) on a dry basis.

#### 4.4.4. Steady Flow Behavior

A starch suspension (1% d.b., *w*/*v*) was prepared in triplicate using deionized water and heated to 90 °C in a water bath for 30 min. Suspensions were cooled down in a water bath until room temperature [62]. A volume of 700 µL of suspension was loaded onto a controlled stress rheometer model CS-50 (Bohlin Instruments, Cranbury, NJ, USA) equipped with a cone–plate geometry (40 mm diameter, 4° cone angle, and 0.15 mm gap). The rheometer was programmed to increase the shear rate from 1 to 200 s^−1^ in 50 s and the experimental data were fitted into a power-law model using Equation (4), where *σ* is the shear stress (Pa), *K* is the consistency coefficient (Pa.s^n^), $\dot{\gamma }$ is the shear rate (s^−1^), and *n* is the flow behavior index (unitless).

(4) $$\sigma =K\times \;{\dot{\gamma }}^{n}$$

#### 4.4.5. Dynamic Oscillation

A starch suspension (8% d.b., *w*/*v*) was prepared in triplicate using deionized water and heated to 90 °C in a water bath for 30 min. Suspensions were cooled down in a water bath until room temperature [62]. The gelatinized starches were loaded onto a controlled stress rheometer model CS-50 (Bohlin Instruments, Cranbury, NJ, USA) equipped with a cone–plate geometry (40 mm diameter and 0.15 mm gap). The mechanical spectra were obtained by frequency sweep from 0.63–62.8 rad/s at a constant strain of 0.5% (within the linear viscoelastic region range). The complex modulus (G*), elastic modulus (*G*′), viscous modulus (*G*″), and complex viscosity (η*), were recorded as function frequency (*ω*) and fitted using Equations (5)–(8), where K*, *K*’, and *K*″ are the consistency coefficients (Pa.s^n^) and n*, *n*′, and *n*″ are the index values (unitless).

(5) $$G^{*}=K^{*}\;\times \;\omega^{n^{*}}$$

(6) $$G^{\prime }=K^{\prime }\;\times \;\omega^{n^{\prime }}$$

(7) $$G^{\prime \prime }=K^{\prime \prime }\;\times \;\omega^{n^{\prime \prime }}$$

(8) $$\eta^{*}=\frac{G^{*}}{\omega }$$

### 4.5. Statistical Analysis

Each sort of test involved the analysis of samples at random. After the Shapiro–Willk test confirmed that the data were normal, a one-way ANOVA was conducted to determine the statistical differences between the starches in all of the data. Data were reported as mean standard deviation, keeping the significant values permitted by the size of the standard deviation, and statistical tests were run using Tukey’s test at a 5% significance level.

## Figures and Tables

**Table 1:** Distribution and particle size characterization of the commercial, *Q. pyrenaica*, and *Q. robur* acorn starches extracted under control and optimum conditions.

Parameter	Commercial	P0.1/t20	P460/t20	R0.1/t17.4	R333/t17.4
<5 μm (%)	6.4 ± 0.0 ^b^	9.3 ± 0.0 ^e^	6.2 ± 0.0 ^a^	8.2 ± 0.0 ^c^	9.0 ± 0.0 ^d^
5–10 μm (%)	12.4 ± 0.0 ^a^	24.5 ± 0.1 ^d^	17.1 ± 0.1 ^b^	23.8 ± 0.0 ^c^	26.1 ± 0.1 ^e^
10–25 μm (%)	79.1 ± 0.0 ^e^	44.0 ± 0.1 ^d^	37.1 ± 0.2 ^a^	37.9 ± 0.0 ^b^	39.0 ± 0.1 ^c^
>25 μm (%)	2.1 ± 0.0 ^a^	22.2 ± 0.2 ^b^	39.6 ± 0.3 ^e^	30.1 ± 0.1 ^d^	25.9 ± 0.3 ^c^
Span (μm)	0.9 ± 0.0 ^a^	3.7 ± 0.1 ^b^	13.0 ± 0.4 ^e^	7.2 ± 0.1 ^d^	6.3 ± 0.2 ^c^
UD (unitless)	0.3 ± 0.0 ^a^	1.4 ± 0.1 ^b^	3.4 ± 0.1 ^e^	2.4 ± 0.1 ^d^	2.0 ± 0.0 ^c^
D_3,2_ (μm)	8.7 ± 0.0 ^c^	8.2 ± 0.0 ^a^	11.3 ± 0.1 ^e^	9.1 ± 0.0 ^d^	8.6 ± 0.0 ^b^
D_4,3_ (μm)	14.0 ± 0.0 ^a^	26.8 ± 0.8 ^b^	73.3 ± 2.6 ^e^	42.3 ± 1.2 ^d^	34.6 ± 0.7 ^c^
D_10_ (μm)	8.1 ± 0.0 ^e^	5.2 ± 0.0 ^a^	6.4 ± 0.0 ^d^	5.5 ± 0.0 ^c^	5.3 ± 0.0 ^b^
D_50_ (μm)	14.0 ± 0.1 ^b^	13.3 ± 0.1 ^a^	18.5 ± 0.1 ^d^	14.2 ± 0.0 ^c^	13.2 ± 0.1 ^a^
D_90_ (μm)	20.9 ± 0.0 ^a^	53.8 ± 1.1 ^b^	246.8 ± 9.0 ^e^	107.3 ± 1.3 ^d^	87.9 ± 3.1 ^c^
SSA (m^2^/kg)	687.2 ± 0.2 ^c^	731.4 ± 1.6 ^e^	533.3 ± 2.2 ^a^	661.2 ± 0.6 ^b^	695.7 ± 2.4 ^d^

P0.1/t20: *Q. pyrenaica* acorn starch extracted under control conditions (0.1 MPa for 20 min); P460/t20: *Q. pyrenaica* acorn starch extracted under optimum conditions (460 MPa for 20 min); R0.1/t17.4: *Q. robur* acorn starch extracted under control conditions (0.1 MPa for 17.4 min); R333/t17.4: *Q. robur* acorn starch extracted under optimum conditions (333 MPa for 17.4 min); UD: uniformity dispersion; SSA: specific surface area. Significant differences between starches are represented by lower-case letters, and values in the same row with the same letters are not significant (*p* > 0.05).

**Table 2:** Characterization of the commercial, and *Q. pyrenaica* and *Q. robur* acorn starches extracted under control and optimum conditions.

Parameter	Commercial	P0.1/t20	P460/t20	R0.1/t17.4	R333/t17.4
Moisture (%)	9.6 ± 0.0 ^a^	13.1 ± 0.3 ^c^	11.5 ± 0.2 ^b^	13.9 ± 0.1 ^d^	16.5 ± 0.2 ^e^
Total solids (%)	90.4 ± 0.0 ^e^	86.9 ± 0.3 ^c^	88.5 ± 0.2 ^d^	86.1 ± 0.1 ^b^	83.5 ± 0.2 ^a^
Damaged starch (%)	1.01 ± 0.03 ^b^	0.56 ± 0.01 ^a^	3.83 ± 0.07 ^c^	0.57 ± 0.01 ^a^	1.09 ± 0.08 ^b^
Amylose (%)	51.1 ± 0.1 ^a^	53.2 ± 0.5 ^b^	67.7 ± 0.0 ^e^	58.2 ± 0.1 ^c^	66.0 ± 0.0 ^d^
Amylopectin (%)	45.0 ± 0.1 ^e^	27.6 ± 0.5 ^d^	19.8 ± 0.0 ^b^	26.1 ± 0.1 ^c^	15.0 ± 0.0 ^a^
Amylose/Amylopectin (unitless)	1.14 ± 0.00 ^a^	1.93 ± 0.05 ^b^	3.41 ± 0.00 ^d^	2.23 ± 0.01 ^c^	4.40 ± 0.00 ^e^

P0.1/t20: *Q. pyrenaica* acorn starch extracted under control conditions (0.1 MPa for 20 min); P460/t20: *Q. pyrenaica* acorn starch extracted under optimum conditions (460 MPa for 20 min); R0.1/t17.4: *Q. robur* acorn starch extracted under control conditions (0.1 MPa for 17.4 min); R333/t17.4: *Q. robur* acorn starch extracted under optimum conditions (333 MPa for 17.4 min); significant differences between starches are represented by lower-case letters and values in the same row with the same letters are not significant (*p* > 0.05).

**Table 3:** Solubility and swelling power values of the commercial, *Q. pyrenaica*, and *Q. robur* acorn starches extracted under control and optimum conditions.

Parameter	T (°C)	Commercial	P0.1/t20	P460/t20	R0.1/t17.4	R333/t17.4
Solubility (%)	50	n.d.	n.d.	n.d.	n.d.	n.d.
	60	0.83 ± 0.07 ^aA^	2.36 ± 0.18 ^aC^	1.60 ± 0.10 ^aB^	1.46 ± 0.19 ^aB^	1.82 ± 0.18 ^aBC^
	70	4.71 ± 0.33 ^bA^	8.97 ± 0.62 ^bC^	6.22 ± 0.35 ^bB^	4.52 ± 0.13 ^bA^	5.07 ± 0.16 ^bAB^
	80	7.58 ± 0.51 ^cA^	11.79 ± 0.06 ^cD^	10.33 ± 0.24 ^cC^	8.79 ± 0.12 ^cB^	7.71 ± 0.02 ^cA^
	90	19.09 ± 0.08 ^dC^	16.16 ± 0.21 ^dA^	16.18 ± 0.26 ^dA^	17.74 ± 0.36 ^dB^	16.53 ± 0.40 ^dA^
	100	22.88 ± 0.12 ^eE^	17.82 ± 0.16 ^eB^	16.66 ± 0.09 ^eA^	19.71 ± 0.14 ^eD^	18.74 ± 0.36 ^eC^
Swelling power (g/g)	50	n.d.	n.d.	n.d.	n.d.	n.d.
	60	2.73 ± 0.11 ^aA^	6.40 ± 0.10 ^aB^	6.15 ± 0.02 ^aB^	6.53 ± 0.09 ^aB^	6.52 ± 0.37 ^aB^
	70	8.73 ± 0.04 ^bA^	10.10 ± 0.02 ^bB^	9.05 ± 0.02 ^bA^	8.64 ± 0.21 ^bA^	8.82 ± 0.11 ^bA^
	80	8.82 ± 0.05 ^bA^	12.20 ± 0.09 ^cC^	10.20 ± 0.34 ^cB^	9.99 ± 0.31 ^bB^	10.19 ± 0.37 ^cB^
	90	15.68 ± 0.07 ^cB^	15.71 ± 0.17 ^dB^	13.31 ± 0.04 ^dA^	15.39 ± 0.49 ^cB^	15.79 ± 0.23 ^dB^
	100	19.03 ± 0.42 ^cC^	16.83 ± 0.03 ^eB^	14.28 ± 0.26 ^eA^	16.75 ± 0.60 ^cB^	14.07 ± 0.17 ^eA^

P0.1/t20: *Q. pyrenaica* acorn starch extracted under control conditions (0.1 MPa for 20 min); P460/t20: *Q. pyrenaica* acorn starch extracted under optimum conditions (460 MPa for 20 min); R0.1/t17.4: *Q. robur* acorn starch extracted under control conditions (0.1 MPa for 17.4 min); R333/t17.4: *Q. robur* acorn starch extracted under optimum conditions (333 MPa for 17.4 min); n.d.: not detected. Significant differences between starches are represented by lower-case letters and values in the same row with the same letters are not significant (*p* > 0.05). Significant differences between temperatures are represented by capital-case letters and values in the same column with the same letters are not significant (*p* > 0.05).

**Table 4:** In vitro digestibility characterization of the commercial, *Q. pyrenaica*, and *Q. robur* acorn starches extracted under control and optimum conditions.

Starch Content	Commercial	P0.1/t20	P460/t20	R0.1/t17.4	R333/t17.4
RDS (g/100g SM)	16.6 ± 0.4 ^c^	7.9 ± 0.5 ^a^	21.8 ± 0.5 ^d^	12.3 ± 0.8 ^b^	13.0 ± 1.0 ^b^
SDS (g/100g SM)	46.3 ± 1.9 ^d^	18.2 ± 0.4 ^a^	27.7 ± 1.6 ^b^	38.1 ± 1.9 ^c^	20.4 ± 1.9 ^a^
TDS (g/100g SM)	86.9 ± 0.4 ^d^	40.3 ± 2.9 ^a^	65.9 ± 3.5 ^c^	50.4 ± 1.2 ^b^	49.4 ± 2.4 ^ab^
RS (g/100g SM)	9.2 ± 0.3 ^a^	40.5 ± 1.4 ^d^	21.6 ± 0.2 ^b^	33.9 ± 0.1 ^c^	31.6 ± 1.2 ^c^
TS (g/100g SM)	96.1 ± 0.6 ^b^	80.8 ± 4.3 ^a^	87.5 ± 3.7 ^ab^	84.3 ± 1.1 ^a^	81.0 ± 1.2 ^a^

P0.1/t20: *Q. pyrenaica* acorn starch extracted under control conditions (0.1 MPa for 20 min); P460/t20: *Q. pyrenaica* acorn starch extracted under optimum conditions (460 MPa for 20 min); R0.1/t17.4: *Q. robur* acorn starch extracted under control conditions (0.1 MPa for 17.4 min); R333/t17.4: *Q. robur* acorn starch extracted under optimum conditions (333 MPa for 17.4 min); RDS: rapidly digestible starch; SDS: slowly digestible starch; TDS: total digestible starch; RS: resistant starch; TS: total starch; SM: starchy material. Significant differences between starches are represented by lower-case letters and values in the same row with the same letters are not significant (*p* > 0.05).

**Table 5:** Steady flow and dynamic oscillation characterization of the commercial, *Q. pyrenaica*, and *Q. robur* acorn starches extracted under control and optimum conditions.

Parameter	Commercial	P0.1/t20	P460/t20	R0.1/t17.4	R333/t17.4
***σ***					
*K* (Pa.s^n^)	0.020 ± 0.000 ^a^	0.035 ± 0.001 ^b^	0.020 ± 0.000 ^a^	0.036 ± 0.004 ^b^	0.031 ± 0.001 ^b^
*n* (unitless)	0.859 ± 0.001 ^b^	0.782 ± 0.003 ^a^	0.859 ± 0.001 ^b^	0.780 ± 0.023 ^a^	0.778 ± 0.027 ^a^
***η*** *					
*H* (unitless)	−0.957 ± 0.004 ^a^	−0.927 ± 0.005 ^b^	−0.906 ± 0.007 ^c^	−0.909 ± 0.008 ^c^	−0.895 ± 0.004 ^c^
G* (Pa)	53.5 ± 2.6 ^a^	53.9 ± 5.6 ^a^	60.5 ± 2.3 ^a^	58.2 ± 5.7 ^a^	63.0 ± 11.8 ^a^
***G*** *					
n* (unitless)	0.033 ± 0.002 ^a^	0.071 ± 0.001 ^b^	0.087 ± 0.001 ^c^	0.091 ± 0.000 ^c^	0.098 ± 0.001 ^d^
K* (Pa.s^n^)	328.5 ± 9.7 ^a^	360.7 ± 4.6 ^ab^	373.7 ± 1.9 ^b^	411.3 ± 9.6 ^c^	467.2 ± 11.3 ^d^
***G*** **′**					
*n*′ (unitless)	0.033 ± 0.001 ^a^	0.071 ± 0.001 ^b^	0.086 ± 0.001 ^c^	0.091 ± 0.000 ^d^	0.098 ± 0.001 ^e^
*K*′ (Pa.s^n^)	327.4 ± 9.7 ^a^	358.0 ± 4.7 ^ab^	390.7 ± 6.3 ^bc^	407.7 ± 9.2 ^c^	462.0 ± 12.0 ^d^
***G*** **″**					
*n*″ (unitless)	0.096 ± 0.002 ^a^	0.086 ± 0.008 ^a^	0.126 ± 0.005 ^b^	0.097 ± 0.003 ^a^	0.114 ± 0.001 ^b^
*K*″ (Pa.s^n^)	23.0 ± 1.5 ^a^	42.2 ± 1.3 ^b^	52.5 ± 2.1 ^c^	47.3 ± 4.4 ^ab^	61.1 ± 3.1 ^d^

P0.1/t20: *Q. pyrenaica* acorn starch extracted under control conditions (0.1 MPa for 20 min); P460/t20: *Q. pyrenaica* acorn starch extracted under optimum conditions (460 MPa for 20 min); R0.1/t17.4: *Q. robur* acorn starch extracted under control conditions (0.1 MPa for 17.4 min); R333/t17.4: *Q. robur* acorn starch extracted under optimum conditions (333 MPa for 17.4 min); ***σ***: shear stress; η*: complex viscosity; G*: complex modulus; ***G*′**: elastic modulus; ***G*″**: viscous modulus. Significant differences between starches are represented by lower-case letters and values in the same row with the same letters are not significant (*p* > 0.05).

**Table 6:** Identification of the starch extraction conditions from *Q. pyrenaica* and *Q. robur* under optimum and control extraction conditions.

*Quercus* spp.	Pressure (MPa)	Time (min)	Nomenclature
*Q. pyrenaica*	460	20.0	P460/t20
*Q. robur*	333	17.4	R333/t17.4
*Q. pyrenaica*	0.1	20.0	P0.1/t20
*Q. robur*	0.1	17.4	R0.1/t17.4

## Data Availability

Data will be available upon request to the corresponding author.

## Supplementary Materials

The following supporting information can be downloaded at: https://www.mdpi.com/article/10.3390/gels9090757/s1, Table S1: FTIR and XRD characterization of the commercial starch, *Q. pyrenaica*, and *Q. robur* acorn starches extracted under control and optimum conditions; Table S2: Thermodynamical characterization of the commercial starch, *Q. pyrenaica*, and *Q. robur* acorn starches extracted under control and optimum conditions; Figure S1: Particle size distribution of the commercial starch (black line); *Q. pyrenaica* acorn starch extracted under control conditions (red line; 0.1 MPa for 20 min—P0.1/t20); *Q. pyrenaica* acorn starch extracted under optimum conditions (green line; 460 MPa for 20 min—P460/t20); *Q. robur* acorn starch extracted under control conditions (yellow line; 0.1 MPa for 17.4 min—R0.1/t17.4); *Q. robur* acorn starch extracted under optimum conditions (blue line; 333 MPa for 17.4 min—R333/t17.4); Figure S2: FTIR spectra of the commercial starch (black line); *Q. pyrenaica* acorn starch extracted under control conditions (red line; 0.1 MPa for 20 min—P0.1/t20); *Q. pyrenaica* acorn starch extracted under optimum conditions (green line; 460 MPa for 20 min—P460/t20); *Q. robur* acorn starch extracted under control conditions (yellow line; 0.1 MPa for 17.4 min—R0.1/t17.4); *Q. robur* acorn starch extracted under optimum conditions (blue line; 333 MPa for 17.4 min—R333/t17.4); Figure S3: Mechanical behavior regarding complex viscosity (*η**; circle symbols) and complex modulus (*G**; triangle symbols) of the commercial starch (black color); *Q. pyrenaica* acorn starch extracted under control conditions (red color; 0.1 MPa for 20 min—P0.1/t20); *Q. pyrenaica* acorn starch extracted under optimum conditions (green color; 460 MPa for 20 min—P460/t20); *Q. robur* acorn starch extracted under control conditions (yellow color; 0.1 MPa for 17.4 min—R0.1/t17.4); *Q. robur* acorn starch extracted under optimum conditions (blue color; 333 MPa for 17.4 min—R333/t17.4); Figure S4: Mechanical behavior regarding the elastic (*G′*; diamond symbols) and viscous (*G″*; square symbols) moduli of the commercial starch (black color); *Q. pyrenaica* acorn starch extracted under control conditions (red color; 0.1 MPa for 20 min—P0.1/t20); *Q. pyrenaica* acorn starch extracted under optimum conditions (green color; 460 MPa for 20 min—P460/t20); *Q. robur* acorn starch extracted under control conditions (yellow color; 0.1 MPa for 17.4 min—R0.1/t17.4); *Q. robur* acorn starch extracted under optimum conditions (blue color; 333 MPa for 17.4 min—R333/t17.4).
