# Supplementary material for: Modification of Acorn Starch Structure and Properties by High Hydrostatic Pressure
Source: Gels. 2023 Sep 17;9(9):757. doi: 10.3390/gels9090757 (PMC10529620; doi:10.3390/gels9090757)
Supplement: Supplementary file 1 [file gels-09-00757-s001.zip › gels-2616736-supplementary.pdf]

gels

MDPI

Article

Modification of Acorn Starch Structure and Properties by High Hydrostatic Pressure

[1] CBQF-Centro de Biotecnologia e Química Fina—Laboratório Associado, Escola Superior de Biotecnologia, Universidade Católica Portuguesa, Rua Diogo Botelho 1327, 4169-005 Porto, Portugal; lgcastro@ucp.pt (L.M.G.C.); cpfpereira@ucp.pt (C.F.P.); sdsousa@ucp.pt (S.C.S.); mbrassesco@ucp.pt (M.E.B.); mmachado@ucp.pt (M.M.); oramos@ucp.pt (Ó.L.R.)
[2] LAQV-REQUIMTE—Laboratório Associado, Department of Chemistry, University of Aveiro, 3810-193 Aveiro, Portugal; elisabete.alexandre.pt@gmail.com (E.M.C.A.); jorgesaraiva@ua.pt (J.A.S.)
[3] Department of Chemistry, University of Aveiro, 3810-193 Aveiro, Portugal; icaco@ua.pt
* Correspondence: mpintado@ucp.pt

Supplementary Materials

Table S1. FTIR and XRD characterization of the commercial starch, and *Q. pyrenaica* and *Q. robur* acorn starches extracted under control and optimum conditions.

Analysis	Parameter	Commercial	P0.1/t20	P460/t20	R0.1/t17.4	R333/t17.4
FTIR	1047/1022	1.043 ± 0.003 ^{a}	1.079 ± 0.001 ^{b}	1.055 ± 0.000 ^{a}	1.194 ± 0.007 ^{c}	1.078 ± 0.001 ^{b}
	955/1022	1.081 ± 0.005 ^{a}	1.172 ± 0.001 ^{c}	1.124 ± 0.000 ^{b}	1.446 ± 0.016 ^{d}	1.181 ± 0.002 ^{c}
XRD	DPT	A	C	C	C	C
	RC (%)	29.3 ± 1.3 ^{b}	22.4 ± 1.6 ^{a}	20.1 ± 1.9 ^{a}	20.2 ± 2.3 ^{a}	20.4 ± 1.5 ^{a}

P0.1/t20: *Q. pyrenaica* acorn starch extracted under control conditions (0.1 MPa for 20 min); P460/t20: *Q. pyrenaica* acorn starch extracted under optimum conditions (460 MPa for 20 min); R0.1/t17.4: *Q. robur* acorn starch extracted under control conditions (0.1 MPa for 17.4 min); R333/t17.4: *Q. robur* acorn starch extracted under optimum conditions (333 MPa for 17.4 min); DPT: Diffraction pattern type; RC: Relative crystallinity. Significant differences between starches are represented by lower-case letters and values in the same row with the same letters are not significant ($p > 0.05$).

Table S2. Thermodynamical characterization of the commercial starch, and *Q. pyrenaica* and *Q. robur* acorn starches extracted under control and optimum conditions.

Parameter	Commercial	P0.1/t20	P460/t20	R0.1/t17.4	R333/t17.4
T_o (°C)	67.3 ± 0.5 ^{b}	55.4 ± 0.3 ^{a}	56.1 ± 0.6 ^{a}	55.0 ± 0.4 ^{a}	55.7 ± 0.3 ^{a}
T_p (°C)	72.6 ± 0.5 ^{c}	62.9 ± 0.4 ^{ab}	63.5 ± 0.3 ^{b}	62.3 ± 0.2 ^{ab}	61.6 ± 0.9 ^{a}
T_c (°C)	77.8 ± 0.3 ^{b}	68.6 ± 1.3 ^{a}	66.9 ± 0.6 ^{a}	66.8 ± 0.3 ^{a}	66.9 ± 0.6 ^{a}
ΔH (J/g)	23.2 ± 2.5 ^{b}	12.7 ± 1.5 ^{a}	13.3 ± 0.3 ^{a}	11.9 ± 1.3 ^{a}	11.9 ± 1.3 ^{a}

P0.1/t20: *Q. pyrenaica* acorn starch extracted under control conditions (0.1 MPa for 20 min); P460/t20: *Q. pyrenaica* acorn starch extracted under optimum conditions (460 MPa for 20 min); R0.1/t17.4: *Q. robur* acorn starch extracted under control conditions (0.1 MPa for 17.4 min); R333/t17.4: *Q. robur* acorn starch extracted under optimum conditions (333 MPa for 17.4 min); T_o: Onset temperature; T_p: Peak temperature; T_c: Conclusion temperature; ΔH: Gelatinization enthalpy. Significant differences between starches are represented by lower-case letters and values in the same row with the same letters are not significant ($p > 0.05$).

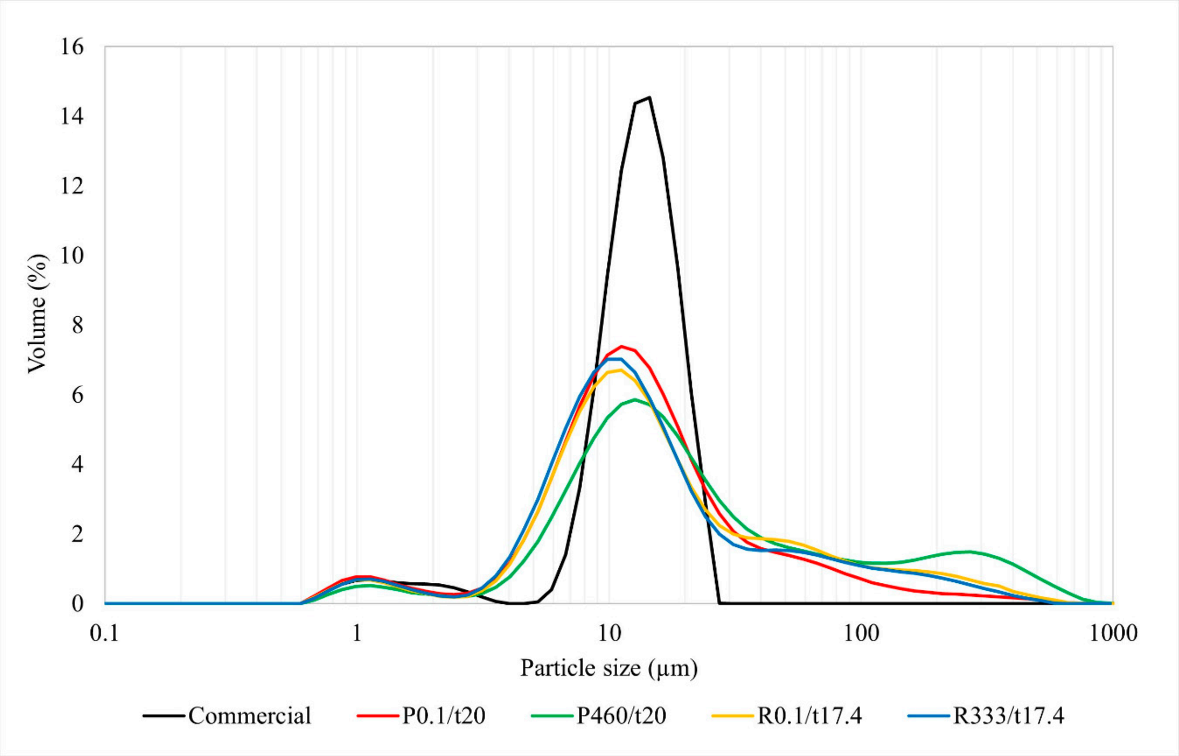

Figure S1. Particle size distribution of commercial starch (black line); *Q. pyrenaica* acorn starch extracted under control conditions (red line; 0.1 MPa for 20 min - P0.1/t20); *Q. pyrenaica* acorn starch extracted under optimum conditions (green line; 460 MPa for 20 min - P460/t20); *Q. robur* acorn starch extracted under control conditions (yellow line; 0.1 MPa for 17.4 min - R0.1/t17.4); *Q. robur* acorn starch extracted under optimum conditions (blue line; 333 MPa for 17.4 min - R333/t17.4).

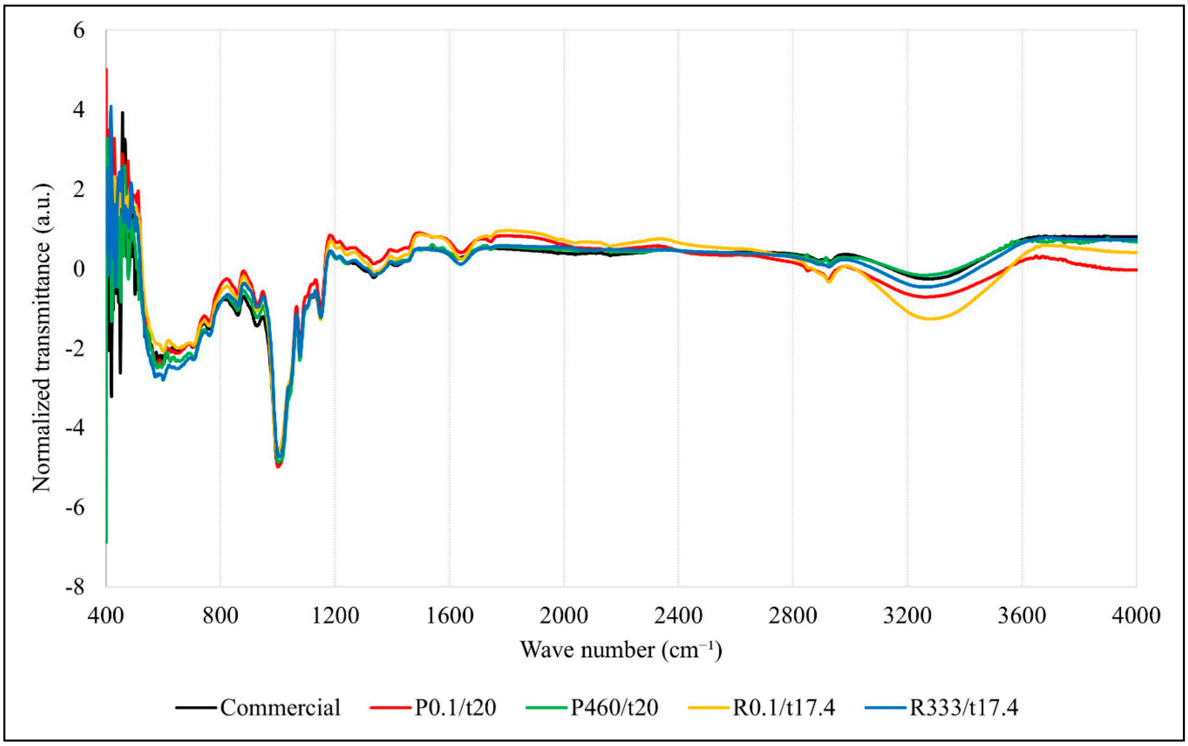

Figure S2. FTIR spectra of the commercial starch (black line); *Q. pyrenaica* acorn starch extracted under control conditions (red line; 0.1 MPa for 20 min - P0.1/t20); *Q. pyrenaica* acorn starch extracted under optimum conditions (green line; 460 MPa for 20 min - P460/t20); *Q. robur* acorn starch

extracted under control conditions (yellow line; 0.1 MPa for 17.4 min - R0.1/t17.4); *Q. robur* acorn starch extracted under optimum conditions (blue line; 333 MPa for 17.4 min - R333/t17.4).

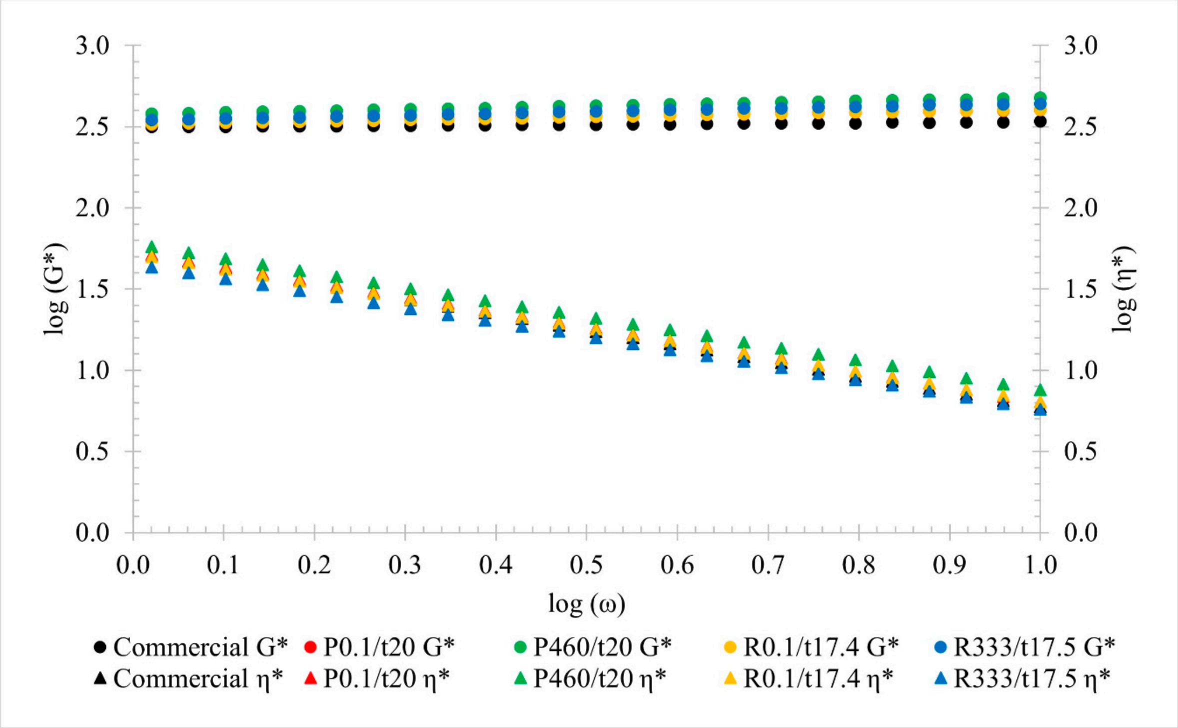

Figure S3. Mechanical behaviour regarding complex viscosity (η*; circle symbols) and complex modulus (G*; triangle symbols) of the commercial starch (black colour); *Q. pyrenaica* acorn starch extracted under control conditions (red colour; 0.1 MPa for 20 min - P0.1/t20); *Q. pyrenaica* acorn starch extracted under optimum conditions (green colour; 460 MPa for 20 min - P460/t20); *Q. robur* acorn starch extracted under control conditions (yellow colour; 0.1 MPa for 17.4 min - R0.1/t17.4); *Q. robur* acorn starch extracted under optimum conditions (blue colour; 333 MPa for 17.4 min - R333/t17.4);.

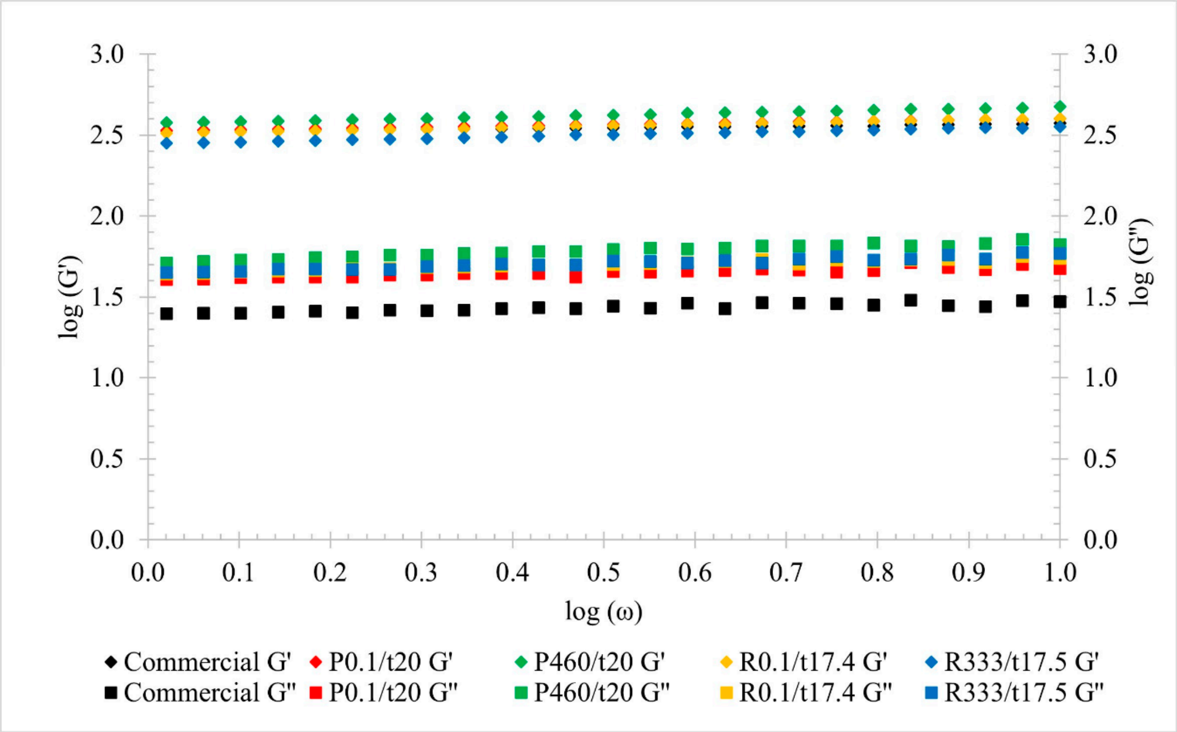

Figure S4. Figure S4: Mechanical behaviour regarding the elastic (G′; diamond symbols) and viscous (G′′; square symbols) of the commercial starch (black colour); *Q. pyrenaica* acorn starch extracted under control conditions (red colour; 0.1 MPa for 20 min - P0.1/t20); *Q. pyrenaica* acorn starch extracted under optimum conditions (green colour; 460 MPa for 20 min - P460/t20); *Q. robur* acorn starch extracted under control conditions (yellow colour; 0.1 MPa for 17.4 min - R0.1/t17.4); *Q. robur* acorn starch extracted under optimum conditions (blue colour; 333 MPa for 17.4 min - R333/t17.4).
